# De Novo Pathogenic Variant in *FBRSL1*, Non OMIM Gene Paralogue *AUTS2*, Causes a Novel Recognizable Syndromic Manifestation with Intellectual Disability; An Additional Patient and Review of the Literature

**DOI:** 10.3390/genes15070826

**Published:** 2024-06-22

**Authors:** Nenad Bukvic, Marta De Rinaldis, Massimiliano Chetta, Antonio Trabacca, Maria Teresa Bassi, René Massimiliano Marsano, Lenka Holoubkova, Maria Rivieccio, Maria Oro, Nicoletta Resta, Jennifer Kerkhof, Bekim Sadikovic, Luigi Viggiano

**Affiliations:** 1 Medical Genetic, Department of Precision and Regenerative Medicine and Ionian Area (DiMePRe-J), University of Bari Aldo Moro, 70124 Bari, Italy; nicoletta.resta@uniba.it; 2 Medical Genetics Section, University Hospital Consortium Corporation Polyclinics of Bari, 70124 Bari, Italy; 3 Unit for Severe Disabilities in Developmental Age and Young Adults, Associazione “La Nostra Famiglia”—IRCCS “E. Medea”, Scientific Hospital for Neurorehabilitation, Piazza A. Di Summa, 72100 Brindisi, Italy; marta.derinaldis@lanostrafamiglia.it; 4 Medical Genetics Laboratory, A.O.R.N. Cardarelli, Building Y, 80127 Naples, Italy; massimiliano.chetta@aocardarelli.it (M.C.); mar.oro94@libero.it (M.O.); 5 Scientific Direction, Scientific Institute IRCCS Eugenio Medea, Via D. L. Monza 20, Bosisio Parini, 23842 Lecco, Italy; antonio.trabacca@lanostrafamiglia.it; 6 Laboratory of Molecular Biology, Scientific Institute IRCCS Eugenio Medea, Via D. L. Monza 20, Bosisio Parini, 23842 Lecco, Italy; mariateresa.bassi@lanostrafamiglia.it; 7 Department of Biosciences, Biotechnology and Environment, University of Bari Aldo Moro, 70125 Bari, Italy; renemassimiliano.marsano@uniba.it (R.M.M.); luigi.viggiano@uniba.it (L.V.); 8 ReStart—Professional Practice of Occupational Therapy, Via di Vittorio, 76125 Trani, Italy; lholoubkova@yahoo.com; 9 Verspeeten Clinical Genome Centre, London Health Sciences Centre, London, ON N6A 3K7, Canada; jennifer.kerkhof@lhsc.on.ca (J.K.); bekim.sadikovic@lhsc.on.ca (B.S.); 10 Department of Pathology and Laboratory Medicine, Western University, London, ON N6A 3K7, Canada

**Keywords:** FBRSL1 and AUTS2, neurodevelopment, polycomb complex

## Abstract

*FBRSL1*, together with *FBRS* and *AUTS2* (Activator of Transcription and Developmental Regulator; OMIM 607270), constitutes a tripartite AUTS2 gene family. AUTS2 and FBRSL1 are evolutionarily more closely related to each other than to FBRS (Fibrosin 1; OMIM 608601). Despite its paralogous relation to AUTS2, FBRSL1’s precise role remains unclear, though it likely shares functions in neurogenesis and transcriptional regulation. Herein, we report the clinical presentation with therapeutic approaches and the molecular etiology of a patient harboring a de novo truncating variant (c.371dupC) in FBRSL1, leading to a premature stop codon (p.Cys125Leufs*7). Our study extends previous knowledge by highlighting potential interactions and implications of this variant, alongside maternal and paternal duplications, for the patient’s phenotype. Using sequence conservation data and in silico analysis of the truncated protein, we generated a predicted domain structure. Furthermore, our in silico analysis was extended by taking into account SNP array results. The extension of in silico analysis was performed due to the possibility that the coexistence of *FBRSL1* truncating variant contemporary with maternal and paternal duplication could be a modifier of proband’s phenotype and/or influence the novel syndrome clinical characteristics. FBRSL1 protein may be involved in neurodevelopment due to its homology with *AUTS2*, together with distinctive neuronal expression profiles, and thus should be considered as a potential modulation of clinical characteristics in a novel syndrome. Finally, considering that FBRSL1 is apparently involved in neurogenesis and in transcriptional regulatory networks that orchestrate gene expression, together with the observation that different genetic syndromes are associated with distinct genomic DNA methylation patterns, the specific episignature has been explored.

## 1. Introduction

The function of the *FBRSL1* gene is largely unknown, and so far, there is no association, with the possible exception of three cases described by Ufartes et al. [1], with any syndromic phenotype. Ufartes and colleagues reported observations in three children with de novo truncating variants of *FBRSL1* (Fibrosin Like 1; Gene ID: 57666, National Library of Medicine, National Center for Biotechnology Information) and exhibited nearly identical clinical presentations. *FBRSL1* was previously identified as a candidate RNA binding protein [2]. *FBRSL1*, together with *FBRS* and *AUTS2* (Activator of Transcription and Developmental Regulator; OMIM 607270), constitutes a tripartite AUTS2 gene family [3]. These paralog genes were probably generated by two rounds of whole genome duplication (R-2WGD) responsible for the evolution of jawed vertebrates and therefore can be defined as a family of ohnolog genes [4]. These ohnologs have been associated with cancer and other genetic disease due to pathogenic copy number alterations and nucleotide variants [5,6,7] (Holland and Short 2008; Dickerson and Robertson 2012; McLysaght et al. 2014). The first description of causative mutations in the *FBRSL1* paralogue *AUTS2* (NG_034133.1) gene (AUTS2 syndrome, OMIM 615834) was reported in 2013 [8]. *AUTS2* and *FBRSL1* are evolutionarily more closely related to each other than to *FBRS* (Fibrosin 1; OMIM 608601) [3]; hence, it is not surprising that *FBRSL1* and *AUTS2* have overlapping phenotypes. Furthermore, the use of animal models indicates that *FBRSL1* and *AUTS2* share common functions in vertebrate development [5].

As previously mentioned, the functions of *FBRS* and *FBRSL1* have yet to be characterized in a neuronal context, but interaction between the Polycomb group proteins *PCGF3* and *PCGF5* with *AUTS2*, *FBRS* and *FBRSL1* have been shown [9]. Namely, AUTS2 and FBRSL1 are components of the Polycomb repressive subcomplexes PRC1.3 and PRC1.5 [10,11,12]. It has been demonstrated that some target genes, such as *HOX* genes and several transcription factors, could be inactivated by Polycomb complexes in a developmental-specific manner during embryogenesis [13,14,15]. Interestingly, inhibition of the repressive function is obtained by binding of AUTS2 to the respective polycomb complex (PRC1.3 or PRC1.5), and thus, the AUTS2-PRC complex acts as a transcriptional activator. This is achieved, as previously demonstrated [10,16], by the recruitment of histone acetyltransferase p300, a known transcriptional co-activator, and casein kinase 2, which inhibits the repressive PRC1 function. However, it remains unknown whether an FBRSL1–PRC complex acts in a repressive or activating manner. On the other hand, *de novo* pathogenic variations in members of PRC1 and PRC2, the same as in trithorax group proteins (which represent the functional counterparts of polycomb complexes), are described in association with malformation and intellectual disability syndromes [17,18,19,20,21,22]. Therefore, it is likely that further uncharacterized PRC-associated proteins, like FBRSL1, are also associated with syndromic pathologies.

Herein, we report the clinical presentation and molecular etiology of a patient with a *de novo* heterozygous truncating variant in *FBRSL1* (c.371dupC). This variant reduces the protein length from 1045 aa (wt Protein Size: 1045 aa; Molecular mass: 110907 Da—Gene Cards, Human Gene Database 2024 https://www.genecards.org/cgi-bin/carddisp.pl?gene=FBRSL1, accessed on 10 February 2024), resulting in a protein of 131 aa (p.Cys125Leufs*7). A predicted domain structure was created by combining sequence conservation data with in silico analysis of the truncated protein. Furthermore, in silico analysis was extended by the implementation of the proband’s SNP array results. Specifically, two maternal duplications (respectively of ~470 Kb on the short arm of the chromosome 16 p13.11 (14866284 bp–15328800 bp) and ~620 kb of ~1.1 Mb on the same chromosome (15417031 pb–16529801 pb)) and a contemporary duplication, of paternal origin (of ~530 Kb on the short arm of the chromosome 7 p11.2 (56118293 bp–56639964 bp)) were evidenced. The extension of in silico analysis was performed due to the possibility that the coexistence of a *FBRSL1* truncating variant contemporary with maternal and paternal duplication could be a modifier of the proband’s phenotype and/or influence the novel syndrome’s clinical characteristics. FBRSL1 protein may be involved in neurodevelopment due to its homology with *AUTS2*, together with distinctive neuronal expression profiles, and thus should be considered as a potential candidate for FBRSL1-associated diseases (the FBRSL1 gene up to today has not been reported as an OMIM gene, and so, no number is assigned to this gene).

Finally, considering that FBRSL1 apparently is involved in neurogenesis and in transcriptional regulatory networks that orchestrate gene expression, together with the observation that different genetic syndromes are associated with distinct genomic DNA methylation patterns, the specific episignature has been tested.

## 2. Materials and Methods

### 2.1. Patient Recruitment

The patient’s parents provided written informed consent to perform genetic testing, and the full content of this publication was in accordance with the Declaration of Helsinki (1984) and its subsequent revisions, the same as for any other applicable local ethical and legal requirements. Furthermore, this study was approved by the Institutional Review Board Committee at Scientific Institute IRCCS ‘‘E. Medea”—Brindisi Research Centre, Brindisi.

### 2.2. Clinical Exome Sequencing (CES)

Next-generation sequencing analysis was performed on genomic DNA from peripheral venous blood (QIAamp DNA Blood Mini Kit) with a clinical exome sequencing panel kit. Approximately 11 Mb (114,405 exons) of the conserved coding regions that cover >4000 genes were enriched with >150,000 probes, which were designed based on human genome sequences (Sophia Genetics SA, Saint Sulpice, Switzerland). Library preparation and sequencing were performed according to the manufacturer’s protocol on MiSeq Instrument (Illumina, San Diego, CA, USA). The mean depth of coverage was 70×. Raw data were analyzed using SOPHiA™ DDM (Sophia Genetics SA) with algorithms for alignment including single nucleotide polymorphisms (SNPs), insertions/deletions (Pepper™, Sophia Genetics SA patented algorithm), and copy number variations (Muskat™, Sophia Genetics SA patented algorithm). The raw reads were aligned to the human reference genome (GRCh37/hgl9), and an integrative genomics viewer (IGV) was used to visualize the binary alignment map (BAM) files.

### 2.3. SNP Array Analysis

High-resolution SNP array analysis of the proband and his parents was carried out by using the CytoScan HD array (Thermo Fisher Scientific, Waltham, MA, USA) as previously described [23,24].

This array contains more than 2.6 million markers for copy number variations (CNVs) analysis and approximately 750,000 SNP probes capable of genotyping with an accuracy greater than 99%.

Data analysis was performed using the Chromosome Analysis Suite Software version 4.1 (Thermo Fisher Scientific) following a standardized pipeline described in the literature [8]. Base pair positions, information about genomic regions and genes affected by CNVs, and known associated diseases were derived from the University of California Santa Cruz (UCSC) Genome Browser, build GRCh37 (hg19).

### 2.4. 3D Modeling and Protein Interactions

FBRSL1 protein and the truncating protein of 131 aa resulting from de novo heterozygous variant p.Cys125Leufs*7 were modeled using I-TASSER (Iterative Threading ASSEmbly Refinement https://zhanglab.ccmb.med.umich.edu/I-TASSER/, accessed on 10 February 2024), a hierarchical approach to define protein 3D structure and function prediction.

The analysis also allows detection of the presence of possible structural domains and predicts protein–ligand binding sites.

The *.pdb files generated from I-TASSER were loaded and visualized on ChemDraw software (version 8; Cambridge Software; PerkinElmer, Inc., Waltham, MA, USA).

### 2.5. STITCH In Silico Analysis

The study was expanded to include the CGH array data, merging the two duplications of unknown importance of the maternal and paternal origin genes using the Sequence To Interaction Thread (STITCH), to explore molecular interactions within biological systems. STITCH elucidates intricate networks governing cellular processes and disease mechanisms, providing a score for each interaction using four types of evidence, i.e., experimental validation, manually curated databases, text mining, and predicted interactions.

### 2.6. Methylation Studies

Methylation analysis was performed with the clinically validated EpiSignTM assay as previously described [25,26]. Briefly, methylated and unmethylated signal intensity generated from the EPIC array was imported into R 3.5.1 for normalization, background correction and filtering. β values ranging from 0 (no methylation) to 1 (complete methylation) were calculated as a measure of methylation level and processed through the established support vector machine (SVM) classification algorithm for EpiSign disorders. The EpiSign knowledge database, composed of over 10,000 methylation profiles from reference disorder-specific and unaffected control cohorts, was utilized by the classifier to generate disorder-specific methylation variant pathogenicity (MVP) scores. MVP scores are a measure of prediction confidence for each disorder, ranging from 0 (discordant) to 1 (highly concordant). A positive classification typically generates MVP scores greater than 0.5; in combination with the assessment of hierarchical clustering and multidimensional scaling, these scores are used in generating the final matched EpiSign result.

## 3. Results

### 3.1. Patient’s Clinical History

**Figure 1:**
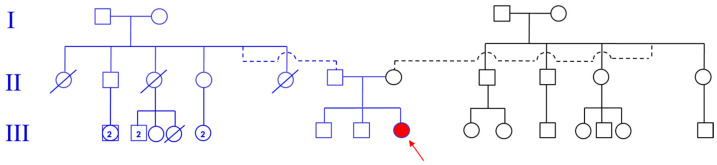
“Pedigree” of index patient (III_11_).

Family history was unremarkable, especially for neurodevelopmental disorders, brain abnormalities, recurrent miscarriages, other birth defects and/or genetic illnesses. The proband was the third child born of a healthy non-consanguineous Italian couple (see pedigree—Figure 1). In prenatal history, factors to be reported include threatened miscarriage occurring in the first and second trimester of gestation and poor fetal movements. At the time of birth, her father and mother were 46 and 38 years old, respectively.

**Figure 2:**
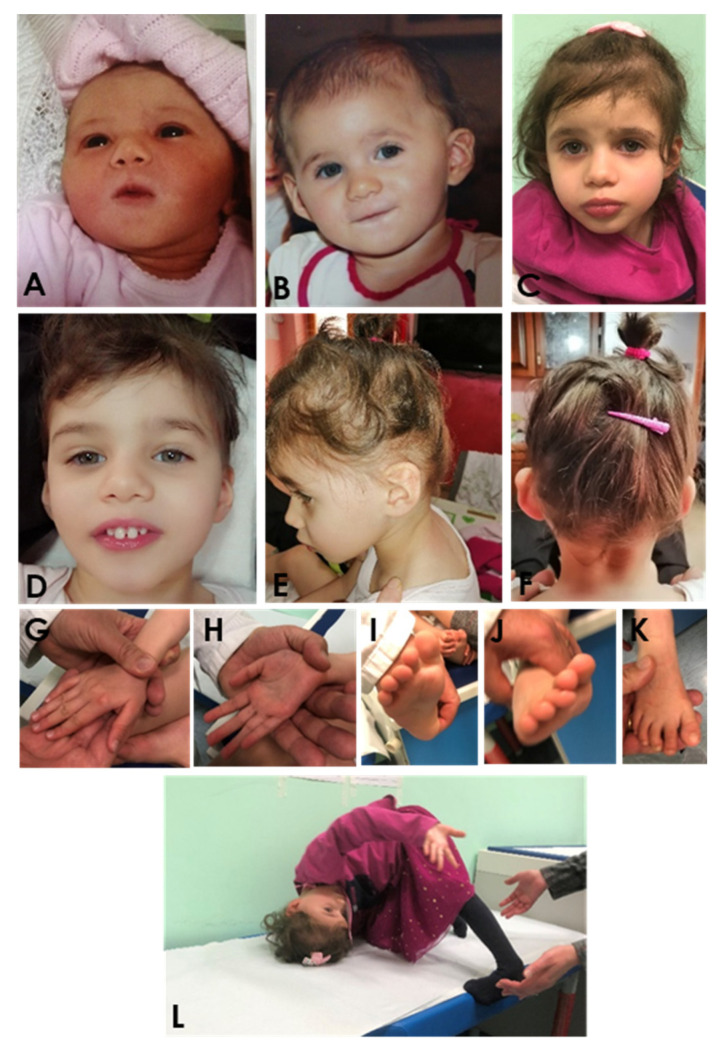
Patient at different periods of life: ~1 month (**A**), ~6 months (**B**) and ~48 months (**C**–**L**).

The child was born spontaneously at full term with no asphyxia, with APGAR scores 8/9 at 1’/5’, respectively. Her birth weight was 2860 g (<10th centile), length 39.5 cm (<3rd centile), head circumference 34 cm (<50th centile). She had neonatal feeding difficulties, due to weak sucking, and postnatal growth retardation. The following abnormalities of the skeletal system were observed: bilateral contractures on hands and fingers; bilateral congenital dislocation of the hip; femurs, radius and ulnas manifested distal irregularities of bone growth front, while tibias and fibulas manifested proximal and distal anomaly of bone growth front. Furthermore, global developmental delay was observed: she gained head control at 7 months of age and seat without support at 14 months of age; she walked without support at 46 months of age. At 48 months of age, she did not develop active speech, and she showed severe intellectual disability (her developmental quotient was 22 as tested with Griffiths Mental Development Scales). No behavioral disorders, such as autistic spectrum disorders, were found, but she developed simple motor stereotypies (head nodding, trunk rotating) and complex arm and hand stereotypies. At the age of 48 months, her height was 96 cm (−1.34 SD below 3rd centile) and her weight was 11.4 kg (−2.68 SD below 3rd centile); she showed also microcephaly (head circumference was 45.3 cm, −2.68 SD bellow 3rd centile) and facial dysmorphism (flat back of the head, frontal bossing, mid-face hypoplasia, short philtrum, micrognathia, and microstomia with “whistling mouth” (Figure 2). The patient keeps standing with help, and wide-based walking with poor balance was observed.

**Figure 3:**
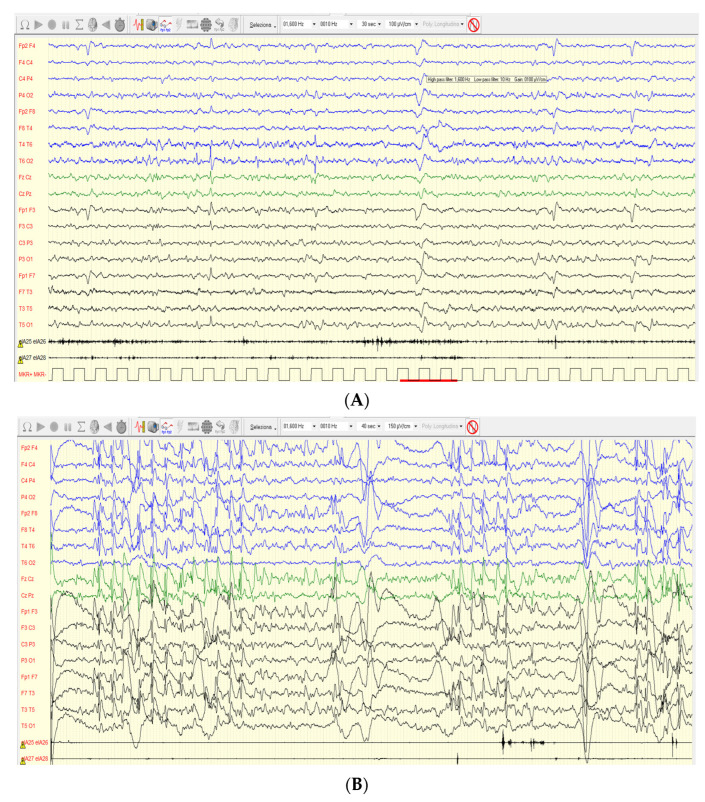
EEG (**A**) Awake polygraphic (bilateral deltoid muscles) video EEG, recorded with the internationally standardized 10–20 system: ictal EEG underlined in red shows diffuse high-voltage slow-wave complex followed by short voltage attenuation prevalent on both anterior regions. No real change in deltoids muscle tone. (**B**) Sleep polygraphic (bilateral deltoid muscles) interictal EEG, recorded with the internationally standardized 10–20 system, showed 2 Hz burst of spikes and spike-and-wave complexes prevalent on the bilateral fronto-central regions and midline.

The neurological evaluation was performed with the observation of mild scoliosis, the same as slight hypertonia of the distal part of the limbs (legs) with supinated and varus feet. Audiology assessment was normal, and no heart defect was found. At the age of 46 months, epileptic attacks were registered with daily drop tonic seizures, lasting a few seconds, single and in runs, in most cases during wakefulness and without fall. Valproic acid (VPA) was administered early with immediately good control of seizures, but months later, the patient underwent a relapse with daily drop tonic seizures and myoclonic absences, so ethosuximide and rufinamide were added to valproic acid with better but not complete control. Drop tonic ictal EEG showed a high-voltage slow wave followed by voltage attenuation (Figure 3A). Interictal EEG showed abnormal sleep architecture and the presence of frequent spikes and spike-and-wave complexes on the bilateral fronto-central regions, the same as midline region, and brief 2 Hz diffuse spike-and-wave complexes (Figure 3B). Myoclonus of the extremities was also observed without a clear correlation with paroxysmal EEG activity.

Cerebral and spinal magnetic resonance imaging (MRI) at 2 months and 4 years of age were normal.

Metabolic evaluations (urinary mucopolysaccharides, urinary oligosaccharides, urinary amino acids, urinary organic acids, plasma amino acids, plasma sphingolipids) were negative.

At the age of 4 years and 4 months, the patient with her parents passed through our Genetic Ambulatory Counselling, where physical examination revealed head circumference of 46.5 cm (−1.1 SD below 3rd centile), body weight of 12.5 kg (<3rd centile) and a body height of 100 cm (>5th <10th centile).

The patient was noted to have facial dysmorphic features (small forehead, bitemporal narrowing, eyebrows, sunken eyes, broad nasal bridge, short philtrum, micrognathia, particular earlobes (medial part of antihelix and antitragus were laterally exposed, almost “convex”; helix from Darwin’s tubercle to the correspondence of antitragus was straight), thick lips and others—Figure 2) with microcephaly, short stature, intellectual disability with no active speech, growth retardation, and cerebral palsy/spasticity. Finally, during the visit on a few occasions, the patient assumed a particular position (as reported in Figure 2 L), and at the age of 6 years, a percutaneous endoscopic gastrostomy (PEG) procedure for enteral feeding was performed due to well-known feeding difficulties and poor weight gain.

Based on objective examinations, hypothetical extensive diagnoses were made, and genetic tests were carried out, taking into consideration that previously performed SNP array (SNPa) results could not explain the patient’s clinical situation.

Peripheral blood samples were collected from the patient and parents after obtaining written informed consent. Trio clinical exome sequencing (CES) was performed on blood DNA samples of the patient and her parents using Agilent’s SureSelectXT Human All Exon V7 Enrichment method on an Illumina NextSeq 500 sequencer. The sequenced reads were aligned to reference target regions, and variants were called with the BWA enrichment application (which also includes GATK for variant calling) available on BaseSpace Onsite (Illumina, San Diego, CA, USA). ANNOVAR was used for annotation against the RefSeq and Single Nucleotide Polymorphism (dbSNP) databases. The filtering strategy we applied led us to select only variants located in the coding regions, including splice sites, that exhibited a minor allele frequency of <1% or were not present in variant databases, including the 1000 Genomes Project and The Genome Aggregation Database (GnomAD). Synonymous variants were excluded. Before CES, an SNP array analysis had been performed.

**Figure 4:**
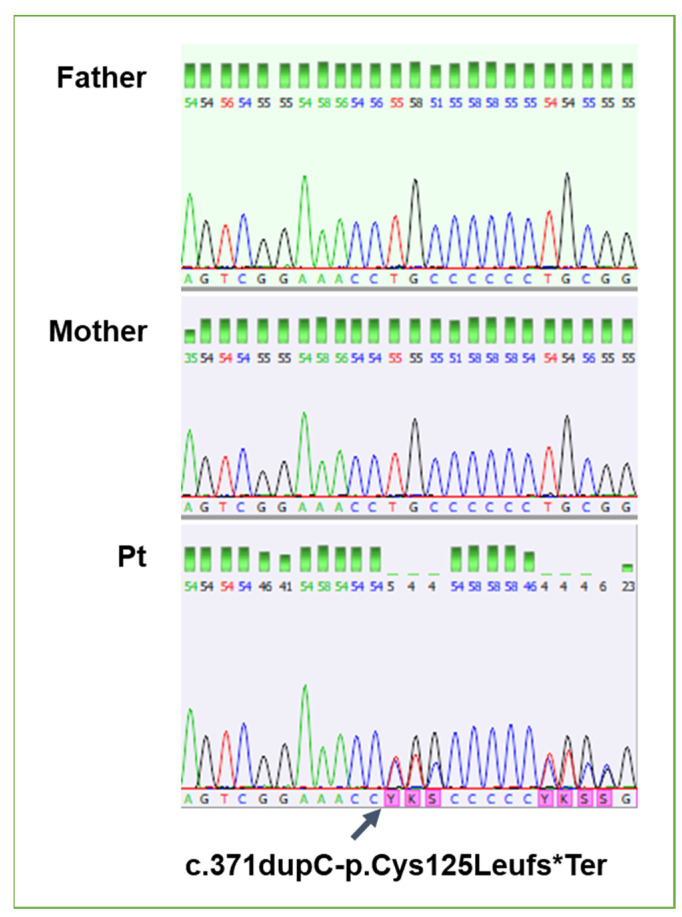
Confirmation of the de novo variant in the proband by Sanger sequencing, and comparison of electropherograms between father, mother, and proband.

Trio clinical exome sequencing (CES) showed in exon 2 of the FBRSL1gene a *de novo* heterozygous variant, duplication: c.371dupC (NM_001142641.2), causing frameshift and leading to a premature stop codon (p.Cys125Leufs*7)—(Figure 4). This variant was not reported in the scientific literature. No additional *de novo* variants or other variants explaining the child’s symptoms were detected by the exome sequencing.

SNP array analysis had been performed previously with subsequently reported results: one paternal duplication, ~530 Kb in 7p11.2 (56118293–56639964), and two maternal duplications: the first of ~470 Kb in 16p13.11 (14866284–15328800) and a second one of ~1.1 Mb in 16 p13.11 (15417031–16529801). All three microduplications (2 mat and 1 pat, respectively) were considered as variants of uncertain significance (VUS). The other family members refused the genetic test.

### 3.2. Methylation Studies

EpiSignTM is a clinically validated, genome-wide DNA methylation assay that enables detection of episignatures, epigenetic biomarkers that can be used for diagnostic screening and functional characterization of genetic variants in a growing number of genetic disorders [25,26]. The EpiSignTM v3 assay has been used to screen for over 50 conditions with known DNA methylation episignatures. Episignature analysis can be used as a functional assessment for variant pathogenicity by comparing the DNA methylation profile of the patient with those from reference-affected and unaffected control cohorts to generate disorder-specific methylation variant pathogenicity (MVP) scores. EpiSign assessment of the patient revealed a genome-wide DNA methylation profile that aligned with negative controls across all episignatures evaluated, including the one associated with the polycomb repressive complex 2 (PRC2), as indicated by an MVP score of zero. Currently, FBRSL1 or PRC1.5 episignatures are undefined and therefore unavailable for assessment.

### 3.3. Rehabilitation Therapy

The patient was referred to rehabilitation due to her initial diagnosis of arthrogryposis multiplex congenita at age 4 months, and is turning up today. She initially presented with joint movement limitations, hand and foot deformities, hip dysplasia, general hypotonicity of her trunk, sucking/feeding difficulties and cognitive delays.

Initial assessment and intervention were based on frameworks, models and approaches that took into account the patient’s diagnosis and included the following: the normal development frame of reference, biomechanical frame of reference, frame of reference for motor skill acquisition, compensatory frame of reference, neuro-developmental treatment frame of reference and sensory integration frame of reference. Therapeutic intervention included functional stimulation and facilitation of motor developmental patterns, strengthening and stretching exercises, splinting, functional orthotics and casts for prevention of further deformity, passive kinesiotherapy, sensory stimulation, cognitive stimulation, hydrotherapy, and hippo therapy. The patient’s family was included as an integral part of her therapeutic team and was consistently educated in correct handling, feeding strategies, play stimulation and cognitive stimulation.

A rehabilitative approach was followed, and as the patient achieved developmental milestones and therapy goals, therapeutic goals was adapted and graded to ensure age- and ability-appropriate, as well as cost-effective intervention was provided. Therapy intervention was part of a multidisciplinary and transdisciplinary approach, which focused on achieving age-appropriate goals with shared transdisciplinary techniques to ensure the carryover and consistency of patient-specific care. Some of the transdisciplinary strategies used by all team members included an augmentative and alternative communication system, which assists the patient in her communication. To ensure compliance and participation, Bing, a cartoon character, was used to facilitate communication, self-regulation, understanding emotions and overall interaction. It is important to note that Bing is a character that the patient is very interested in and assists greatly in directing attention and focus as well as providing emotional support through challenging activities.

The patient achieved most of her development milestones and is now able to sit independently, stand independently, reach, grasp, release and manipulate objects of interest with her upper limbs and hands, and walk with minimal support. She is able to communicate her basic needs, comprehend and understand instructions and interact with her environment.

The future therapy plan and goals will follow the same rehabilitative approach and frameworks to ensure normal development of gross and fine motor, cognitive, sensory and neurodevelopmental skills. One of the current main short-term goals is to integrate the patient into a school setting where she will be able to advance her ability and to ensure that she experiences a feeling of belonging and achievement. Her participation in continuous therapeutic input and placement in a school setting, with adaptation and support, will ensure that she will become a valuable part of society and live a rewarding life.

## 4. Discussion

Truncating variants in specific exons of Fibrosin-like protein 1 (FBRSL1) were recently reported in three unrelated children with a previously unknown malformation syndrome. The clinical spectrum includes postnatal microcephaly, no active speech, facial dysmorphism, cleft palate, skin creases, skeletal anomalies and contractures, postnatal growth retardation, global developmental delay as well as respiratory problems, hearing impairment, and heart defects [1,27]. The clinical phenotype of this novel malformation syndrome caused by FBRSL1 variants partially overlaps with the severe form of AUTS2 syndrome (Table 1).

However, our patient reported clinical characteristics basically comparable with those of the three patients reported by Ufartes et al. (2020), and at the age of 46 months manifested epileptic attacks. Therapy with Valproic acid (VPA) was administered early and good control of seizures has been obtained.

FBRSL1 (NC_000012.12, Fibrosin Like 1) is currently a poorly characterized gene and is a paralogue of AUTS2 (NC_000007.14, Activator of Transcription and Developmental Regulator).

Both genes synthesize two proteins that are part of the Polycomb subcomplexes PRC1.3 and PRC1.5, which have an important function during embryonic development by inactivating different target genes at specific developmental time points. Although it is not clear how PRC1.3 and PRC1.5 are recruited to chromatin, studies suggest that several DNA-binding transcription factors may be involved.

PCGF3 (Polycomb Group Ring Finger Protein 3; OMIM 617543) and PCGF5 (Polycomb Group Ring Finger Protein 5; OMIM 617407) share a group of interactors, including AUTS2, FBRS, FBRSL1, WDR68, and CK2, but the role of FBRSL1 remains unknown.

In the present report, we analyzed a child with a de novo heterozygous truncating variant in the *FBRSL1* gene (c.371dupC) that produces a protein of 131 aa (p.Cys125Leufs*7). Compared to the validated FBRSL1 primary long isoforms (NM_015174.3 and NM_001284303.2), these isoforms differ in their splicing, which can affect their function and localization within the cell. Isoform 1 is often considered the primary form, used in various studies related to neural and craniofacial development, while isoform 2 may have specialized or context-specific roles [27].

Shorter N-terminal isoforms of FBRSL1 are particularly significant. They have been shown to localize both in the nucleus and the cytoplasm, suggesting diverse functional roles. These isoforms are crucial for normal development, as demonstrated in studies where their absence or mutation led to severe defects. For instance, in Xenopus laevis, N-terminal isoforms were necessary for heart development and craniofacial formation [27]. FBRSL1 isoforms play critical roles during embryogenesis. Knockdown experiments in animal models such as Xenopus laevis have demonstrated that disrupting these isoforms leads to developmental abnormalities, including craniofacial defects and impaired heart development. In particular, the short N-terminal isoform has been shown to rescue developmental defects caused by gene knockdown [28].

No other variants that may be related to clinical characteristics have been identified by exome sequencing.

The analysis was carried out taking into account that several short N-terminal FBRSL1 isoforms presented a co-localization with the centrosomes and the kinetochores, suggesting a role in the microtubule-kinetochore organization, as well as possible appropriate cell division control with consequences for neurogenesis regulation and embryonic CNS growth.

**Figure 5:**
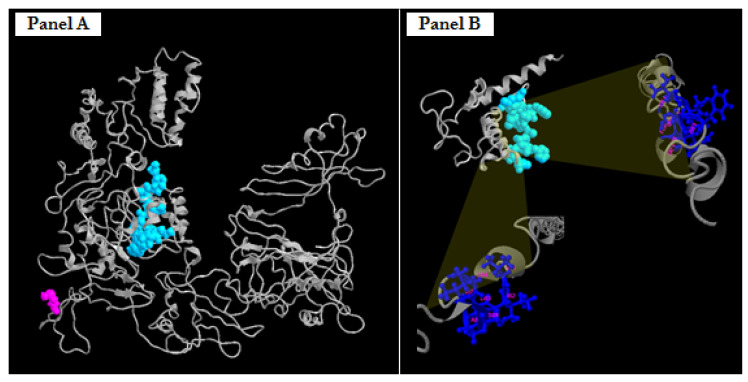
Predicted three-dimensional structures of FBRSL1, wild-type (panel **A**) and truncated proteins p.Cys125Leufs*7 (panel **B**). Highlighted in light blue space-filling mode is the predicted protein domain, which extends from the amino acid residues 81 to 93 capable of binding the amino acid residues of FBRSL1 in the same position. Highlighted in blue ball-and-stick mode are the specific amino acid residues (at the top right, 85, 86, 87, 90, 91, 92, 93 aa; on the bottom left, 81, 82, 83, 84, 87, 88, 89 aa) able to bind the light blue domain.

The in silico analysis (Figure 5) of the truncated protein of 131 aa revealed a specific protein domain that extends from the amino acid residues 81 to 93 capable of binding the amino acid residues of FBRSL1 in the same position.

It has been demonstrated (1) that the protein FBRSL1 is localized in both nuclear and extranuclear subcellular regions, depending on its isoforms. On the basis of this evidence, we assumed that the presence of the peptide, although small in size, may have an effect on the possible localization of the protein, by virtue of the fact that it retains the possible binding domain (acid residues 81 to 93).

**Figure 6:**
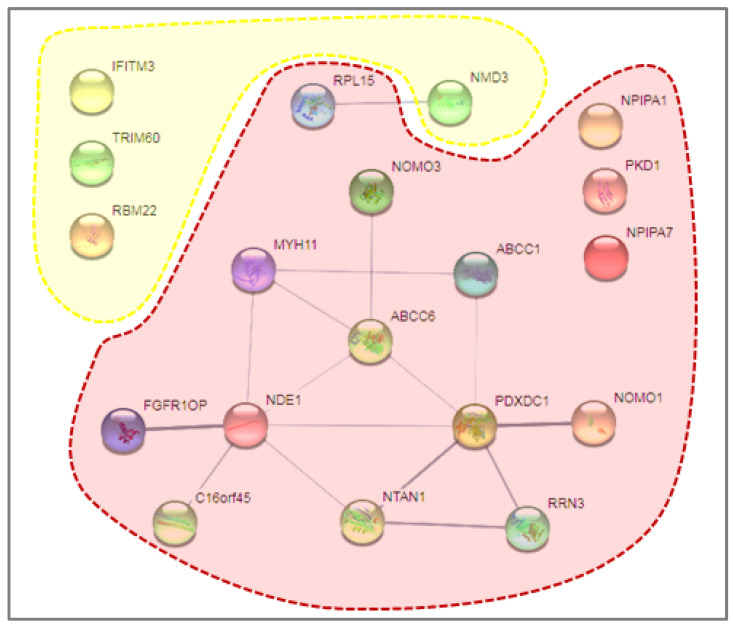
STITCH in silico analysis. Emphasized in yellow are the proteins involved in paternal duplication, while in red, the proteins involved in maternal duplication. Only a possible relationship between paternal protein NMD3 and maternal protein RPL15 was detected, as highlighted in the dotted rectangle.

Different FBRSL1 isoforms have specific subcellular localizations, and this allows one to hypothesize a different function for each isoform. For example, short N-terminal isoforms show a nuclear and cytoplasmatic localization pattern [1,27]. The truncated version found in the patient, which shows two nuclear localization signals, could localize in the nucleus, and thanks to the self-binding domain formed by amino acid residues 81–93, interact with the wild-type isoforms of FBRSL1, inactivating them. This could confer a possible negative dominance effect on the mutation in our case. The analysis was then extended to the results obtained by SNPa, mainly because the proband presented the coexistence of two (VUS) duplications of maternal origin in 16 p13.11, and contemporarily another duplication of paternal origin, also considered as VUS, in 7 p11.2. Due to these results, our idea was to “check-up” by in silico approach if any particular interactions attributable to variation in the combined expression of a possible dysregulation of maternal and paternal proteins were expressed in duplicate regions. This analysis, performed on STITCH, did not recognize any particular interactions attributable to variation in the combined expression except for a possible relationship between paternal protein NMD3 (NMD3 Ribosome Export Adaptor) and maternal protein RPL15 (Ribosomal Protein L15) (Figure 6).

Both proteins are related to the Ribosomal 60S subunit; in fact, the first one is involved in the passage of the 60S subunit through the nuclear pore complex and into the cytoplasm, while the second is a member of the L15E family of ribosomal proteins and a component of the 60S subunit. However, neither of these could be consequently traced back to the clinical phenotype or to a particular symptomatology of our patient.

**Figure 7:**
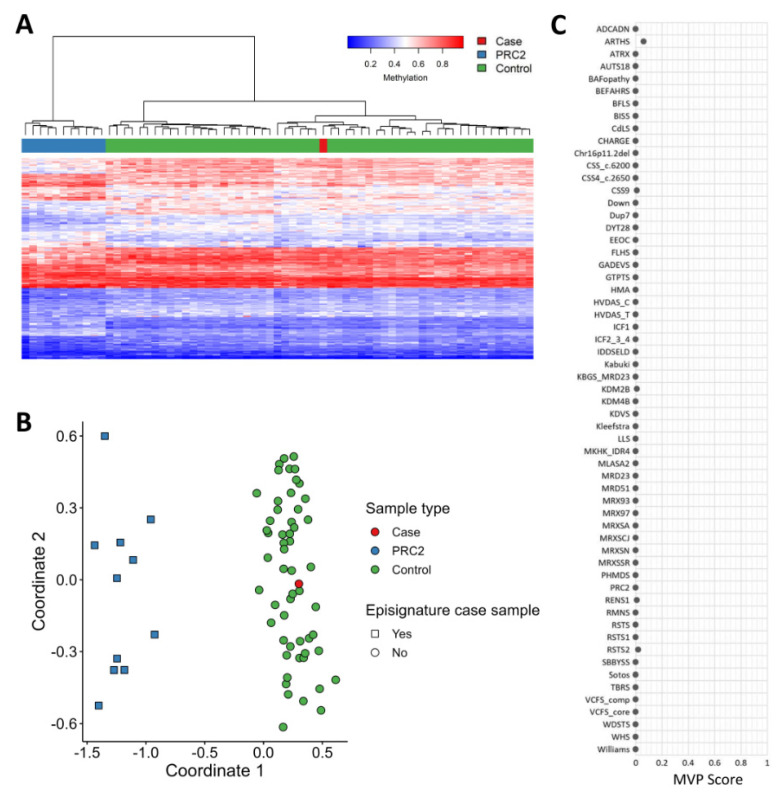
EpiSign (DNA methylation) analysis of peripheral blood from a patient with a truncating *FBRSL1* variant. (**A**) Hierarchical clustering and (**B**) multidimensional scaling plots indicate the patient (red) has a DNA methylation signature similar to controls (green) and distinct from cases with the PRC2 episignature (blue). (**C**) MVP score, a multi-class supervised classification system capable of discerning between multiple episignatures by generating a probability score for each episignature. The lack of elevated scores indicated the patient was similar to controls for all episignatures evaluated.

Finally, it has to be considered that the *FBRSL1* gene (as well as *AUTS2*) encodes for proteins that are part of the Polycomb subcomplexes (PRC1.3 and PRC1.5), which have essential roles in developmental processes by regulating gene expression via histone modification [29]. We decided to perform the clinical implementation of genome-wide DNA methylation analysis (EpiSign) to test the assessment and clinical impact on our proband/patient (Figure 7), but the lack of elevated MVP scores indicated the case was similar to controls for all episignatures evaluated.

However, further functional studies are required to better understand the effects, on molecular processes, of possible pathogenic variants that can be traced back to specific phenotypic characteristics. Follow-up of the patient, reported in Figure 2, which represents our patient in different periods of time, provided more significant insight into the range of phenotypic expressivity.

It is therefore evident that through the use of a multidisciplinary approach that involves the use of molecular and clinical diagnosis reverse phenotyping approaches, a personalized diagnostic workflow, and reviewed literature, a better definition of clinical diagnostic and potential therapeutic approaches to this new neurodevelopmental syndrome is possible.

## Figures and Tables

**Table 1:** Comparison of clinical features seen in three patients with FBRSL1 mutation reported by Ufartes et al. (2020) and our patient.

Characteristics	Ufartes et al., 2020 [1]	Our Patient
**Growth and feeding**		
Low birth weight	2/3	+
Short stature	3/3	+
Microcephaly	3/3	+
Feeding difficulties	3/3	+
**Neurodevelopmental disorders**		
Intellectual disability	3/3	+
Autism/autistic behavior	3/3	+
**Neurological disorders**		
Cerebral palsy/spasticity	2/3	+
Other: respiratory insufficiency with ventilation therapy	3/3	−
**Dysmorphic features**		
High arched eyebrows	2/3	−
Epicanthal folds	2/3	−
Prominent nasal tip	1/3	−
Deep/broad nasal bridge	2/3	+
Short/upturned philtrum	2/3	+
Micro-/retrognatia	1/3	+
Low set ears	2/3	−
Narrow mouth	1/3	−
Other: widely spaced teeth	2/3	+
**Skeletal disorders**		
Kyphosis/scoliosis	3/3	+
Other (camptodactyly/contractures)	3/3	+
**Congenital malformation**		
Patent foramen ovale/atrial septum defect	2/3	−
**Other**		
Cleft palate	2/3	−
Asplenia	1/3	−
Anal atresia	1/3	−
Abnormality of the skin	2/3	−
Hearing impairment	2/3	−
**Other previously not reported**		
Epilepsy		+
Stereotypy (ritualistic movement, posture, or utterance)		+
Dislocation of the hips		+
Mastication problems		+
Percutaneous Endoscopic Gastrostomy (PEG)		+

## Data Availability

The participants of this study did not give written consent for their data to be shared publicly, so due to the sensitive nature of the research supporting data is not available.
